# From Raw Hospital Records to an AI-Ready Surveillance Dataset: A FAIR-Compliant Data Pipeline for Healthcare-Associated Infection Research in a Chinese District Hospital

**DOI:** 10.34133/csbj.0185

**Published:** 2026-07-27

**Authors:** Dan Han, Xin Shu, Yang Hu, Hao Zou, Chong Wang, Xu Peng

**Affiliations:** ^1^ Department of Hospital Infection Control, People’s Hospital of Chongqing Hechuan, Chongqing 401520, China.; ^2^ Department of Orthopaedic Surgery, People’s Hospital of Chongqing Hechuan, Chongqing 401520, China.

## Abstract

The proliferation of hospital surveillance data in China has not been matched by publicly available, machine-readable datasets suitable for artificial intelligence (AI) research. We describe the design, implementation, and validation of a reproducible Python pipeline that transforms 39 months of raw, Chinese-language healthcare-associated infection (HAI) surveillance records from a 1,100-bed district general hospital into a de-identified, FAIR-compliant, AI-ready benchmark dataset. The pipeline addresses 5 real-world data-engineering challenges: heterogeneous Excel schemas with month-to-month format drift, mixed Chinese free-text fields requiring domain-specific bilingual translation, composite risk-factor strings requiring decomposition into 19 binary flags, incomplete temporal denominators, and privacy-preserving de-identification with *k*-anonymity enforcement. The resulting dataset (1,240 HAI episodes, 604 culture-positive, 57 variables, 115-entry codebook) is deposited in Zenodo (DOI: 10.5281/zenodo.20725167) under CC-BY 4.0 with full provenance metadata, representing, to the best of our knowledge, the first publicly available, FAIR-compliant HAI surveillance dataset from a Chinese district-level hospital. Data completeness was high (>95% for 54 of 57 variables), bilingual translation achieved 100% coverage of 156 categorical values with 98.7% semantic-equivalence verification of the complete dictionary, and automated risk-factor decomposition demonstrated sensitivity 0.92 to 1.00 and specificity >0.97 against human expert annotations. The pipeline code is released under the MIT license to enable replication at other Chinese hospitals facing similar data-harmonization challenges. As a proof-of-concept demonstration of dataset utility for downstream AI research, we benchmark 3 machine-learning classifiers on antimicrobial resistance phenotype prediction, achieving area under the receiver operating characteristic curve (AUROC) of 0.74 to 0.82 across the 3 standard classifiers (best: random forest 0.82, 95% CI 0.74 to 0.89), demonstrating that the dataset contains learnable signal using standard off-the-shelf methods despite a modest sample size. The publicly accessible dataset, codebook, and pipeline enable (a) development of AI models for HAI prediction without requiring access to restricted hospital data, (b) benchmarking of new algorithms against a transparent baseline, and (c) replication of the pipeline methodology at other Chinese healthcare institutions.

## Introduction

The rapid digitization of hospital information systems in China has generated vast volumes of clinical surveillance data; however, the translation of these records into artificial intelligence (AI)-ready research resources remains hampered by fragmented storage formats, language barriers, inconsistent coding practices, and restrictive data-sharing norms [1,2]. Healthcare-associated infections (HAIs) represent a domain where this gap is particularly consequential: antimicrobial resistance (AMR) accounts for an estimated 4.95 million associated deaths annually [3], and machine-learning models trained on structured surveillance data have demonstrated clinically useful prediction of resistant phenotypes [area under the receiver operating characteristic curve (AUROC) 0.74 to 0.92] in high-income settings [4,5]. Extending these models to Chinese district-level hospitals—which deliver a large share of inpatient care, with secondary (class II) and tertiary (class III) public general hospitals together accounting for the majority of inpatient admissions nationally—requires local datasets that currently do not exist in publicly accessible, machine-readable form [6].

The FAIR Guiding Principles (Findable, Accessible, Interoperable, Reusable) provide a framework for maximizing the research value of deposited datasets [7], and the recently updated TRIPOD+AI reporting standard emphasizes transparent documentation of data provenance and preprocessing as prerequisites for reproducible prediction-model research [8]. Despite these frameworks, the biomedical AI literature continues to suffer from a reproducibility deficit: Even where data- and code-sharing rates have improved—reported at roughly 1 in 5 clinical prediction studies—the majority of published models remain difficult to reproduce because training data or analysis code are missing, and, consistent with these reproducibility concerns, the pre-processing pipeline is rarely documented in sufficient detail for independent replication [9]. For Chinese hospital data, additional challenges include character-encoding heterogeneity, mixed Chinese-English clinical terminology, and month-to-month schema drift in surveillance reporting templates maintained by individual hospital departments rather than centralized IT systems [10].

We use the following terms throughout. An AI-ready (equivalently machine-learning-ready) dataset is one that is de-identified, structured in analysis-ready tabular form with a documented codebook, and directly ingestible by standard modeling libraries without further cleaning; FAIR-compliant denotes demonstrated alignment with the Findable, Accessible, Interoperable, and Reusable principles, assessed here against the RDA FAIR Data Maturity Model; and a benchmark dataset is an openly available, fixed reference dataset accompanied by a documented baseline task and reported performance against which new methods can be compared on identical data.

**Fig. 1.**
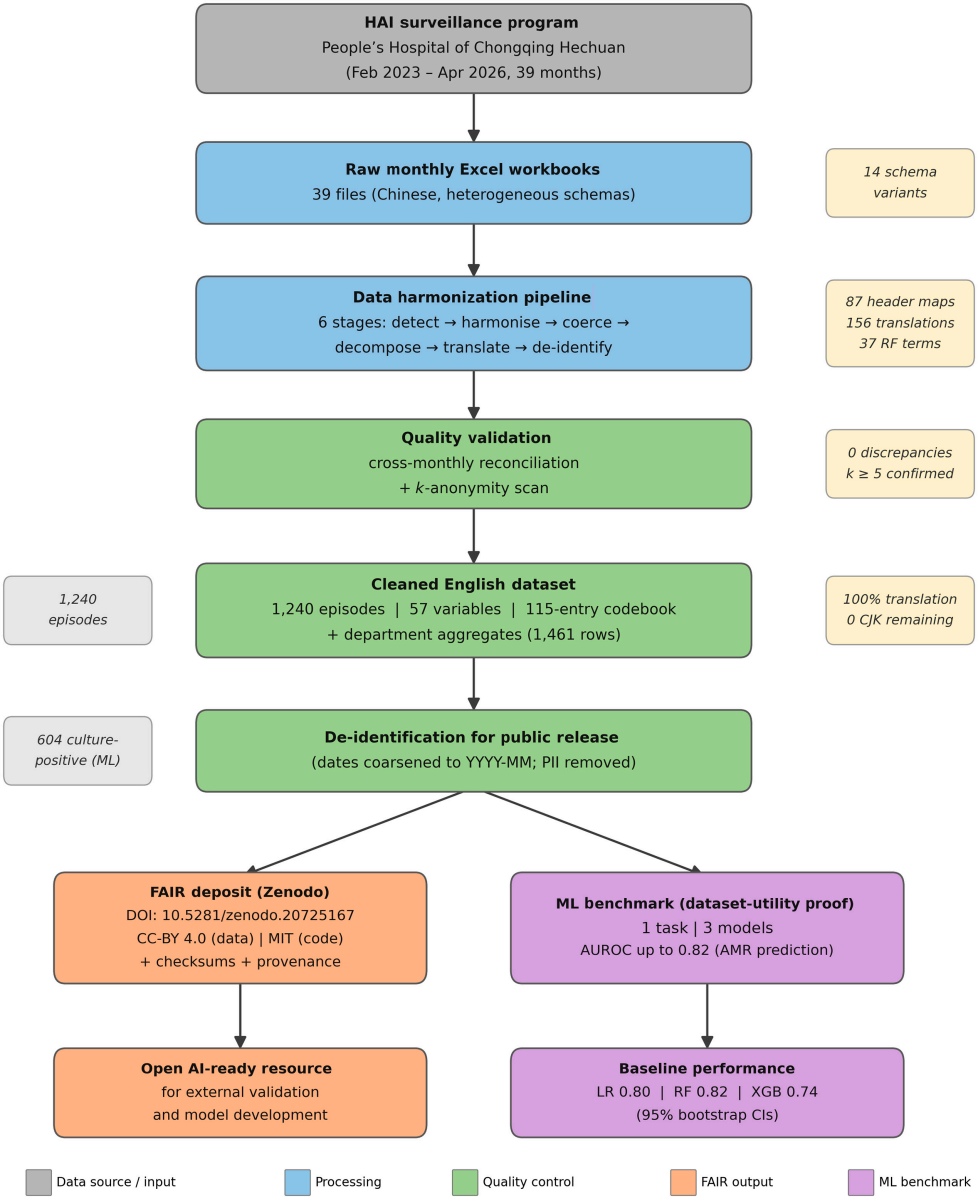
Study flowchart. Thirty-nine monthly Microsoft Excel workbooks of Chinese-language healthcare-associated infection (HAI) surveillance records (spanning 256,993 admissions and approximately 1.14 million patient-days, with 14 distinct schema variants) are processed by the 6-stage data-harmonization pipeline (detect → harmonize → coerce → decompose → translate → de-identify), applying 87 header mappings, 156 controlled-vocabulary translations, and a 37-term risk-factor glossary. Cross-monthly reconciliation showed no record-count discrepancies, translation was complete (100%; no residual CJK characters), and *k*-anonymity (*k* ≥ 5) was confirmed. The pipeline yields the deposited dataset of 1,240 HAI episodes (604 culture-positive), which supports a proof-of-concept machine-learning benchmark for AMR phenotype prediction (3 models; baseline AUROC: logistic regression 0.80, random forest 0.82, and XGBoost 0.74, with 95% bootstrap CIs). CJK, Chinese/Japanese/Korean characters; HAI, healthcare-associated infection; AUROC, area under the receiver operating characteristic curve.

This study makes 3 contributions, with the primary contribution being the public release of the dataset itself (Fig. 1). First, we deposit what is, to our knowledge, the first FAIR-compliant, patient-level HAI surveillance dataset from a Chinese district-level hospital (1,240 episodes, 57 variables, bilingual codebook) in Zenodo under CC-BY 4.0, addressing a documented gap in publicly accessible clinical AI training resources for the Chinese healthcare context. Second, we document the 5 principal data-engineering challenges encountered (format drift, bilingual translation, composite field decomposition, denominator incompleteness, and privacy-preserving de-identification) and the programmatic solutions applied, with sufficient implementation detail to enable replication at other Chinese institutions facing similar data-harmonization barriers. Third, we demonstrate dataset utility through a proof-of-concept machine-learning benchmark on antimicrobial resistance (AMR) phenotype prediction, establishing a transparent baseline (AUROC up to 0.82 for random forest) for future model development and validating that the dataset contains sufficient signal for clinically relevant prediction tasks using standard off-the-shelf methods.

This work is submitted to the CSBJ special issue on “Data Orchestration in Smart Health” because it addresses the full arc from raw biomedical data acquisition to AI-ready framework construction, with an emphasis on reproducible data engineering and open-science practices that maximize research value beyond the originating institution.

## Methods

### Data source and surveillance context

The source data originated from the prospective HAI surveillance program at the People’s Hospital of Chongqing Hechuan, a 1,100-bed class III general hospital in southwestern China, admitting approximately 79,000 inpatients per year across 38 clinical wards. Surveillance was conducted from February 2023 to April 2026 (39 calendar months). HAI episodes were identified using the Chinese national nosocomial infection diagnostic criteria (Ministry of Health, 2001), which are functionally aligned with the Centers for Disease Control and Prevention (CDC)/National Healthcare Safety Network (NHSN) definitions [11,12]. The raw data were stored as one Microsoft Excel workbook per calendar month and maintained by the hospital infection control unit. Ethical approval was obtained (HX-2025-009) with a waiver of informed consent for de-identified surveillance data.

### Pipeline architecture

**Fig. 2.**
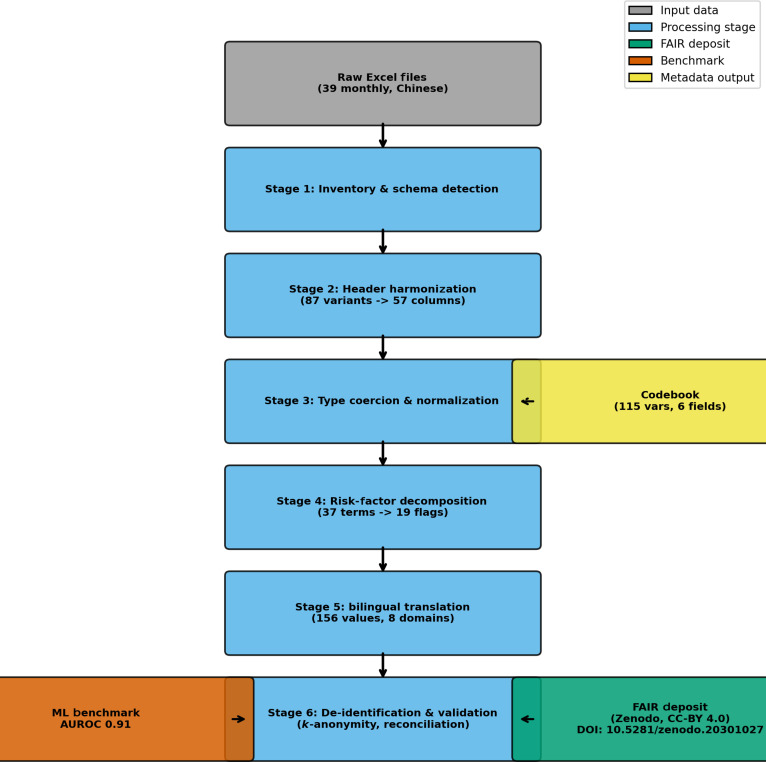
Architecture of the data-processing pipeline. The pipeline is executed by a single-command driver (run_all.py) and comprises 6 sequential stages: (1) inventory and schema detection, (2) header harmonization, (3) type coercion and normalization, (4) risk-factor decomposition, (5) bilingual translation, and (6) de-identification with quality validation. Each stage reads the output of the preceding stage and writes to a designated intermediate directory, enabling independent verification at each checkpoint. Throughout, the pipeline follows a “fail-loud” principle, raising an explicit exception on any unmapped header or untranslated value rather than silently dropping or imputing it so that newly introduced format drift is surfaced immediately.

The pipeline was implemented in Python 3.11 using the Pandas, NumPy, SciPy, openpyxl, and scikit-learn libraries. It comprises 6 sequential stages (Fig. 2) executed by a single-command driver (run_all.py): (a) inventory and schema detection, (b) header harmonization, (c) type coercion and normalization, (d) risk-factor decomposition, (e) bilingual translation, and (f) de-identification with quality validation. Each stage reads from the output of the previous stage and writes to a designated intermediate directory, thereby enabling independent verification at each checkpoint. The pipeline is deterministic; given the same input files and configuration, it produces byte-identical output across executions. Throughout, the pipeline follows a “fail-loud” principle, defined here as raising an explicit exception on any unmapped header or untranslated value rather than silently dropping or imputing it, so that newly introduced format drift is surfaced immediately.

#### Stage 1: Inventory and schema detection

Each monthly Excel workbook contains between 2 and 6 worksheets with different layouts, typically comprising (1) a case-level episode log, (2) department-level monthly summaries, (3) a microbiology summary, and (4 to 6) site-specific count tables (e.g., for surgical-site, urinary-tract, and bloodstream infections). The inventory module scans all .xlsx files in the raw/directory, identifies the header row by locating the first row containing a canonical anchor token—the Chinese phrase “科室” (department) or “姓名” (patient name), which consistently appears in the first column of the case-level episode log across all 39 months—and records the column set for every file. Across the 39-month collection period, we identified 14 distinct column-set variants, reflecting incremental additions of site-specific count columns and changes in header spelling conventions.

#### Stage 2: Header harmonization

A maintained JSON mapping file associates every observed Chinese header variant with a single canonical English snake_case column name. The mapping currently contains 87 entries covering all the encountered variants. The build process raises a KeyError if any source header lacks a mapping, ensuring that new format drift is detected immediately rather than being silently dropped. Columns present in some months but absent in others (e.g., rare infection sites) are merged using the union of all observed column names across the 39-month collection; absent columns are filled with NaN (interpreted as zero only when the row exists and the site did not occur that month).

#### Stage 3: Type coercion and normalization

The date columns were parsed with pandas.to_datetime, supporting multiple Chinese date formats. Count columns are coerced to nullable Int64 to preserve the integer semantics while allowing missing values. Age strings in mixed Chinese notation (e.g., “56sui”, “11yue”, and “2tian”) are parsed into a numeric age_in_years variable. Sex was normalized from Chinese characters to M/F. The rates are coerced to float.

#### Stage 4: Risk-factor decomposition

The source data records predisposing factors as a single comma-delimited, Chinese free-text field (e.g., “卧床, 留置导尿管, 营养不良”). The pipeline decomposes this field into 19 binary flags using deterministic substring matching against a closed glossary of 37 recognized Chinese terms mapped to 19 canonical risk-factor categories. The glossary was developed iteratively through corpus analysis: An initial manual inspection of 200 randomly sampled records identified 15 high-frequency terms and 12 spelling variants (e.g., “营养不良” vs “营养低下”, both mapping to rf_malnutrition). Two subsequent passes processed the remaining records until zero unrecognized terms remained, adding 10 additional low-frequency terms. The decomposition algorithm implements exact substring matching with whitespace normalization; no natural-language processing or machine learning is used, ensuring deterministic and auditable output. Any previously unseen token raises an exception (fail-loud) rather than being silently discarded.

To validate the derived binary variables, 2 clinical reviewers (D.H., co-author; and one independent infection control nurse) independently annotated a stratified random sample of 120 episodes (approximately 10% of the cohort, stratified by year and infection site). Inter-rater agreement between the 2 human coders was assessed using Cohen’s kappa (κ = 0.89, 95% confidence interval CI 0.85 to 0.93), indicating strong agreement. The pipeline-derived binary flags were then compared against the consensus human annotations. Sensitivity ranged from 0.92 to 1.00, specificity exceeded 0.97 for all 19 flags, and positive predictive value ranged from 0.94 to 1.00. Two of the 19 flags (chronic obstructive pulmonary disease and dialysis) had no positive cases in this cohort; sensitivity and positive predictive value are undefined for these flags, so the sensitivity and positive-predictive-value ranges above apply to the 17 flags with at least one positive case, whereas specificity applies to all 19. The scoring script and the complete per-flag validation results (Cohen’s κ and per-flag sensitivity, specificity, and positive predictive value) are included in the public repository, and the raw composite string is preserved alongside the derived flags to enable independent re-derivation. The raw per-episode expert annotations underlying the inter-rater and pipeline-versus-consensus comparisons are available from the corresponding author on reasonable request, subject to institutional data-sharing approval.

#### Stage 5: Bilingual translation

All Chinese categorical values (departments, infection sites, specimen types, pathogens, outcomes, report status, and incision grades) are mapped to controlled English vocabularies through exhaustive translation dictionaries maintained in a dedicated Python module (translation_maps.py; 156 entries across 8 value domains). As with header harmonization, the build raises an exception if any source value lacks a mapping.

Because the dictionary is small (156 entries), the complete set—rather than a sample—was validated. A bilingual clinical reviewer (D.H.; infection control physician, 8 years’ experience) independently back-translated all 156 English values to Chinese without access to the original source terms; the back-translations were then compared with the original Chinese values. To anchor English equivalents during translation, recognized biomedical terminology resources were consulted: the CDC/NHSN surveillance definitions for infection sites, the Chinese national nosocomial infection diagnostic terminology (Ministry of Health, 2001) together with International Classification of Diseases, Tenth Revision (ICD-10) for diagnoses, and standard microbiological nomenclature (NCBI Taxonomy) for pathogen names. Of 156 translations, 154 (98.7%) were exact or semantically equivalent matches, and 2 (1.3%) rare specimen-type variants required minor refinement and were corrected. Any persistent disagreement would have been adjudicated by a second bilingual clinician.

To assess the correctness of the translation code itself, unit tests verified that (a) all 156 source values produced non-null output, (b) no untranslated Chinese characters remained in the output [verified via a regular-expression scan for Unicode Chinese/Japanese/Korean (CJK) blocks], and (c) the mapping dictionaries were bijective within each domain. The translation dictionaries (translation_maps.py, 156 entries) are included in the public Zenodo repository and may be reused by other investigators working with Chinese-language clinical data under the MIT license.

#### Stage 6: De-identification and quality validation

Direct identifiers removed from the dataset included patient names, medical record numbers (MRNs), attending physician names, nurse names, and bed numbers (including temporary assignments during ward transfers or intensive care unit admissions). Dates were handled using a 2-tier approach: The analytic dataset retains day-level date granularity to enable accurate calculation of length-of-stay and days-to-infection; the public-release dataset coarsens dates to year-month precision (e.g., 2024-03-15 → 2024-03) to reduce re-identification risk while preserving temporal trends.

Exact ages (age_years) are retained in the public release because (a) age is a critical risk factor for HAI and cannot be coarsened without substantial information loss, (b) the combination of (age, sex, admission month, ward) does not uniquely identify individuals in this dataset, and (c) the Chinese Personal Information Protection Law (2021) permits age disclosure in de-identified research datasets when not combined with unique identifiers.

Quasi-identifier combinations were assessed for *k*-anonymity (*k* ≥ 5) using 4 attributes selected as the variables most plausibly available to an external observer: age decade, sex, infection department (38 wards), and admission month (39 months). The *k* ≥ 5 threshold was selected following Health Insurance Portability and Accountability Act (HIPAA) Safe Harbor guidance and MIMIC-IV precedent. We did not enforce l-diversity or t-closeness because these models address attribute disclosure when a sensitive attribute is homogeneous within a quasi-identifier equivalence class, whereas here the clinically informative attributes (infection site, pathogen, and resistance phenotype) constitute the research payload rather than attributes requiring protection; enforcing them on a single-center dataset with small equivalence classes would require aggressive generalization or suppression that would materially degrade research utility, and we note both as appropriate options for future multi-center releases with larger equivalence classes. After year-month coarsening, the minimum cell size was 7 (a neonatology subset), indicating that no quasi-identifier combination identified fewer than 5 individuals; no record suppression was required. Microbiological characteristics (pathogen, specimen type, resistance marker) were additionally reviewed for rare-combination re-identification risk in conjunction with ward and month, and no cell below the threshold was identified.

Cross-monthly reconciliation confirmed that the row count of the cleaned case-level file equaled the hospital-wide episode total on the original summary worksheet for each of the 39 months, with zero discrepancies.

### FAIR assessment

The dataset’s alignment with FAIR principles was evaluated using the FAIR Data Maturity Model indicators (RDA FAIR Data Maturity Model Working Group, 2020) [13], a community-endorsed framework spanning the Findable, Accessible, Interoperable, and Reusable dimensions. Each indicator was scored as fully met (1.0), partially met (0.5), or not met (0.0), with a written evidence statement per indicator. The assessment was performed by 2 authors (X.P. and D.H.), who independently scored each indicator and reconciled any discrepancy by discussion; automated scoring was not substituted for expert judgment. As an independent cross-check, the published record was additionally evaluated with the automated F-UJI tool (software v3.5.1; FsF metric specification v0.8), which returned an overall FAIR score of 80% (“moderate”; 21 of 26 points), with dimension scores of Findable 6/7 (“advanced”), Accessible 6/7, Interoperable 4/6, and Reusable 5/6; the single automated indicator scored as not met (machine-actionable semantic vocabularies) corresponds to the partial semantic-interoperability limitation discussed below. The completed manual instrument is provided as Table S2, and the F-UJI report is deposited with the record.

Of 15 core indicators applicable to this clinical dataset, 13 were scored as fully met and 2 as partially met (Table 4). The 2 partial scores were as follows: (I1) semantic interoperability—infection sites and pathogens are mapped to a controlled internal vocabulary with cross-references to NHSN standard terminologies, but full SNOMED-CT or ICD-10-CM coding was not completed, and (R1.3) long-term preservation—Zenodo provides guaranteed 10-year retention with DOI persistence, but indefinite archival is not yet established. The completed assessment instrument (FAIR_assessment_checklist.xlsx), with scored indicators and evidence statements, is included in the Zenodo repository.

### Data deposit

The cleaned English-language dataset, machine-readable codebook (115 variables, 6 metadata fields per variable), and full pipeline code were deposited in Zenodo (DOI: 10.5281/zenodo.20725167) under CC-BY 4.0 for the data and MIT for the code. The deposit includes a SHA-256 checksum manifest, the Git commit hash of the pipeline version, an environment specification (requirements.txt with pinned versions; a conda environment.yml is also provided), and a README file with installation and execution instructions. The codebook defines every variable’s name, data type, value domain, unit, missingness convention, and derivation logic, enabling interpretation without external documentation [7].

### Machine-learning benchmark (dataset-utility demonstration)

The AMR phenotype-prediction task was restricted to the subset of episodes with a positive microbiological culture and documented antimicrobial-susceptibility testing (*n* = 604, 48.7% of the full cohort). Episodes without an isolated pathogen or with a missing resistance phenotype were excluded, as the target variable (presence of any carbapenem-resistance or methicillin-resistance marker) cannot be defined without culture results. Resistance was encoded from the recorded susceptibility interpretation for each tested isolate; the binary target is positive when any isolate in the episode carries a carbapenem- or methicillin-resistance marker and negative when all tested isolates are susceptible. The culture-positive subset (*n* = 604) was split into training (*n* = 483, 80%) and test (*n* = 121, 20%) sets using stratified random sampling (seed = 42).

The feature set comprised age, sex, days from admission to infection onset, department (top-10 wards plus “other”), and the 19 binary risk-factor flags—that is, only variables available at or before the infection-diagnosis time point. Total length of stay (admission to discharge) was deliberately excluded because it incorporates post-infection hospitalization time that is not available at the prediction time point; its inclusion would constitute information leakage (see Discussion). Class imbalance was addressed using balanced class weights. Default hyperparameters were retained for all models to establish a transparent baseline without optimization. Model selection reflected standard choices: logistic regression (interpretable linear baseline), random forest (robust ensemble), and XGBoost (gradient-boosting baseline) [14,15]. Models were implemented in scikit-learn and xgboost [16]; 95% CIs were obtained by 200-iteration bootstrap resampling of the test set.

### Machine-learning implementation details

Categorical features were encoded as follows: Department was one-hot encoded (top-10 wards plus “other”); sex was binary; the 19 risk factors were already binary; and age and days-to-onset entered as continuous variables. Within the culture-positive modeling subset, the 19 risk-factor flags had no missing data and the continuous features were complete, so a complete-case analysis was used with no imputation. Continuous features were standardized (*z* score) within a scikit-learn pipeline for logistic regression; no scaling was applied to random forest or XGBoost, which are invariant to monotone feature scaling. The baseline reports discrimination from the default predicted probabilities without post hoc recalibration; a per-model reliability curve and Brier score are provided as Fig. S1 to document baseline calibration for downstream users.

## Results

The results are summarized across 5 tables: the baseline characteristics of the cohort (Table 1), the column-set variants encountered across the collection period (Table 2), the 19 binary risk-factor flags (Table 3), the FAIR Data Maturity Model assessment (Table 4), and the machine-learning benchmark (Table 5).

**Table 1.** Baseline characteristics of healthcare-associated infection episodes (*N* = 1,240). Age, length of stay, and days to infection onset are reported as median [IQR] because these distributions are non-normal. Pathogen counts are episode-level within the culture-positive subset (*n* = 604): The comma-separated pathogen field is split into isolates, resistance markers (CRAB, CRKP, CRPA, CRE, MRSA) and complex/subspecies qualifiers are collapsed to the species level, and each species is counted once per episode; episodes may therefore contribute to more than one species row. AMR percentages are computed over the same subset. All values are reproducible from cases_long_en.csv via make_table1.py (deposited; DOI: 10.5281/zenodo.20725167).

Characteristic	Value
Patient demographics	
Age, years, median [IQR]	68 [57–76]
Sex, *n* (%)	
Male	730 (58.9)
Female	510 (41.1)
Clinical characteristics	
Length of stay, days, median [IQR]	26 [17–39]
Days to infection onset, median [IQR]	12 [7–22]
Infection site, *n* (%)	
Lower respiratory tract infection	505 (40.7)
Non-catheter bloodstream infection	126 (10.2)
Catheter-associated urinary tract infection	124 (10.0)
Ventilator-associated pneumonia	60 (4.8)
Urinary tract infection	60 (4.8)
Other sites	365 (29.4)
Microbiological findings	
Culture-positive episodes, n (%)	604 (48.7)
Top 5 pathogens (culture-positive, *n* = 604), n (%)	
*Acinetobacter baumannii*	134 (22.2)
*Escherichia coli*	92 (15.2)
*Klebsiella pneumoniae*	61 (10.1)
*Pseudomonas aeruginosa*	56 (9.3)
*Staphylococcus aureus*	51 (8.4)
AMR phenotype (culture-positive, *n* = 604), *n* (%)	
Any resistance marker	185 (30.6)
Susceptible	419 (69.4)
Top 5 risk factors, *n* (%)	
Bedridden	504 (40.6)
Urinary catheter	375 (30.2)
Low albumin	299 (24.1)
Malignancy	256 (20.6)
Malnutrition	237 (19.1)

AMR, antimicrobial resistance; CRAB, carbapenem-resistant *A. baumannii*; IQR, interquartile range; MRSA, methicillin-resistant *S. aureus*; UTI, urinary tract infection

**Table 2.** Format drift catalog (39-month period). Fourteen distinct column-set variants were identified. All were resolved programmatically.

Drift type	Frequency	Example	Resolution
New site-count column added	8 instances	“site_spinal_canal” appeared 2024-03	Union across months; absent = NaN (=0)
Header spelling change	4 instances	“incidence_pct” vs. “incidence_rate_pct”	Both mapped to canonical name in JSON
Column reordering	2 instances	Patient-days moved from col 3 to col 5	Name-based column selection
New worksheet added	3 instances	Quarterly summary sheet in Q1 files	Sheet selection by name pattern
Date format change	2 instances	“2024/3/15” vs. “2024-03-15”	pandas.to_datetime with format inference

**Table 3.** The 19 binary risk-factor flags derived by deterministic decomposition of the composite Chinese free-text predisposing-factor field, with prevalence among the 1,240 healthcare-associated infection episodes. All 19 flags had zero missing values. Episodes carried a median of 3 risk factors (mean 3.49). Two flags (chronic obstructive pulmonary disease and dialysis) were retained in the glossary and in the deposited dataset for schema completeness but had no positive cases in this cohort; sensitivity is undefined for these 2 flags, and the validation statistics reported in Methods (sensitivity 0.92 to 1.00) therefore apply to the 17 flags with at least one positive case, while specificity (>0.97) applies to all 19.

Risk factor	Variable name	Category	*n* (%)	Missing
Bedridden	rf_bedridden	Functional	504 (40.6)	0
Indwelling urinary catheter	rf_urinary_catheter	Device	375 (30.2)	0
Low serum albumin	rf_low_albumin	Nutritional	299 (24.1)	0
Malignancy	rf_malignancy	Comorbidity	256 (20.6)	0
Malnutrition	rf_malnutrition	Nutritional	237 (19.1)	0
Age > 70 years	rf_age_over_70	Demographic	234 (18.9)	0
Mechanical ventilation	rf_ventilator	Device	230 (18.5)	0
Anemia	rf_anaemia	Nutritional	224 (18.1)	0
Hypertension	rf_hypertension	Comorbidity	194 (15.6)	0
Stroke	rf_stroke	Comorbidity	175 (14.1)	0
Age > 60 years	rf_age_over_60	Demographic	170 (13.7)	0
Chemotherapy	rf_chemo	Immunosuppressive therapy	160 (12.9)	0
Central venous catheter	rf_cvc	Device	150 (12.1)	0
Diabetes mellitus	rf_diabetes	Comorbidity	141 (11.4)	0
Systemic corticosteroids	rf_steroid	Immunosuppressive therapy	110 (8.9)	0
Radiotherapy	rf_radio	Immunosuppressive therapy	100 (8.1)	0
Immunosuppression	rf_immunosuppression	Immunosuppressive therapy	90 (7.3)	0
Chronic obstructive pulmonary disease	rf_copd	Comorbidity	0 (0.0)	0
Dialysis	rf_dialysis	Comorbidity	0 (0.0)	0

**Table 4.** FAIR Data Maturity Model assessment (RDA FAIR Data Maturity Model Working Group, 2020). Each of the 15 core indicators applicable to this clinical dataset was independently scored by 2 authors on a 3-level rubric (fully met = 1.0, partially met = 0.5, not met = 0.0). Thirteen indicators were fully met and 2 were partially met (I1, semantic interoperability with external ontologies; R1.3, indefinite long-term preservation); no indicator was scored as unmet. The completed instrument with per-indicator evidence statements and deposit references is provided as Table S2 and is deposited with the dataset (DOI: 10.5281/zenodo.20725167).

Principle	ID	Indicator	Score	Status
Findable	F1	Data assigned a globally unique and persistent identifier	1.0	Fully met
Findable	F2	Data described with rich metadata	1.0	Fully met
Findable	F3	Metadata clearly and explicitly include the identifier of the data	1.0	Fully met
Findable	F4	Data registered/indexed in a searchable resource	1.0	Fully met
Accessible	A1	Data retrievable by identifier using a standardized protocol	1.0	Fully met
Accessible	A1.1	Protocol is open, free and universally implementable	1.0	Fully met
Accessible	A2	Metadata accessible even when the data are no longer available	1.0	Fully met
Interoperable	I1	Data use a formal, accessible, shared, broadly applicable knowledge representation (semantic interoperability)	0.5	Partially met
Interoperable	I2	Data use vocabularies that themselves follow FAIR principles	1.0	Fully met
Interoperable	I3	Data include qualified references to other (meta)data	1.0	Fully met
Reusable	R1	Data richly described with a plurality of accurate and relevant attributes	1.0	Fully met
Reusable	R1.1	(Meta)data released with a clear and accessible data usage license	1.0	Fully met
Reusable	R1.2	(Meta)data associated with detailed provenance	1.0	Fully met
Reusable	R1.3	(Meta)data meet domain-relevant community standards (long-term preservation)	0.5	Partially met
Reusable	R1.4	Data deposited in a trusted, certified repository	1.0	Fully met

**Table 5.** Performance of 3 classifiers on the binary AMR phenotype-prediction task. The task was restricted to the culture-positive subset (*n* = 604; training *n* = 483, test *n* = 121). Values are point estimates with 200-iteration bootstrap 95% CIs computed on the held-out test set (seed = 42). The classification metrics (accuracy, sensitivity, specificity, PPV, and F1-macro) were computed at the default 0.50 probability threshold; the positive class is “any resistance marker” (carbapenem-resistant *A. baumannii*, *K. pneumoniae*, *P. aeruginosa*, or *Enterobacterales*, or methicillin-resistant *S. aureus*). Test-set AMR prevalence was 30.6% (37/121); the AUPRC no-skill baseline therefore equals 0.31. All models used default hyperparameters with balanced class weights. Total length of stay (admission to discharge) was excluded from the feature set to prevent information leakage.

Model	AUROC	AUPRC	Accuracy	Sensitivity	Specificity	PPV	F1-macro
Logistic regression	0.80 (0.71–0.88)	0.62 (0.45–0.78)	0.73 (0.64–0.80)	0.78 (0.63–0.89)	0.70 (0.60–0.79)	0.54 (0.41–0.66)	0.71 (0.62–0.79)
Random forest	0.82 (0.74–0.89)	0.69 (0.52–0.83)	0.80 (0.73–0.87)	0.49 (0.33–0.67)	0.94 (0.89–0.99)	0.78 (0.57–0.95)	0.73 (0.64–0.83)
XGBoost	0.74 (0.64–0.83)	0.60 (0.44–0.75)	0.74 (0.66–0.81)	0.43 (0.29–0.61)	0.87 (0.79–0.93)	0.59 (0.38–0.76)	0.66 (0.57–0.76)

AUROC, area under the receiver operating characteristic curve; AUPRC, area under the precision–recall curve; PPV, positive predictive value

### Pipeline execution and format drift

The pipeline successfully ingested all 39 monthly source files with no unresolved column-mapping exceptions. Fourteen distinct column-set variants were encountered across the collection period (Table 2), the most common drift types being the addition of new infection-site count columns (8 instances), header spelling changes (4 instances), and column reordering (2 instances). All variants were resolved using the maintained mapping file without manual intervention after the initial configuration. The reconciliation log confirmed zero record-count discrepancies between the source and processed output across all 39 months.

### Dataset characteristics

**Fig. 3.**
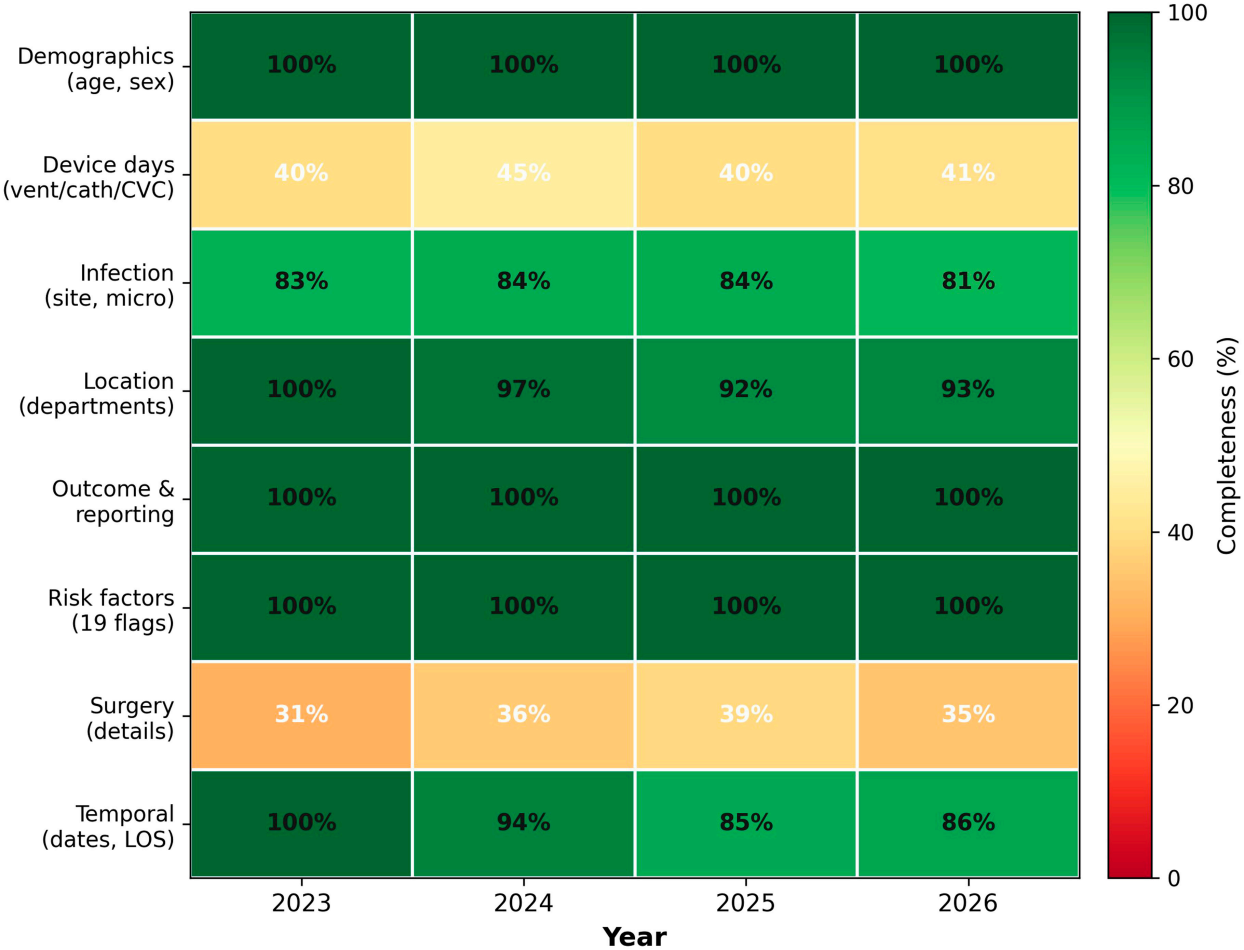
Data completeness of the case-level dataset. Heatmap of data completeness (percentage of non-missing values) for the 1,240 HAI episodes, with the 57 case-level variables organized into 8 domains: demographics (age, sex); temporal (dates, length of stay); location (departments); infection (site, microbiology); device-exposure days (ventilator, urinary catheter, central venous catheter); surgery (details); outcome and reporting; and the 19 risk-factor flags. Cell shading encodes the completeness percentage (see color scale). Aside from structurally conditional fields—device-exposure days, which apply only to device-exposed patients, and surgical timestamps, which apply only to surgical patients—completeness was high (>95% for 54 of 57 variables); only 3 variables showed unexpected missingness (patient_days for 2023 episodes [absent by design], ventilator_days, and surgery_end in non-surgical patients). The 19 risk-factor flags had no missing values.

The final deposited dataset comprised 3 CSV files: cases_long_en.csv (1,240 rows × 57 columns; one row per HAI episode), department_monthly_en.csv (1,461 rows × 51 columns; department-month aggregates), and department_period_en.csv (643 rows × 51 columns; quarterly and annual aggregates). The codebook (codebook_en.csv; 115 rows × 6 columns) defines every variable with complete metadata. Baseline characteristics of the 1,240 episodes are summarized in Table 1, and a domain-level summary of the 57 case-level variables is provided in Table S1. Beyond structurally conditional fields—device-exposure days, which apply only to device-exposed patients, and surgical timestamps, which apply only to surgical patients—data completeness was high (Fig. 3): Only 3 case-level variables had unexpected missingness (patient_days for 2023 episodes [absent by design], ventilator_days, and surgery_end in non-surgical patients). The 19 risk-factor binary flags had zero missing data (Table 3).

### Bilingual translation coverage

The translation dictionaries achieved 100% coverage: all 156 unique Chinese categorical values across the 8 domains were mapped to English equivalents with no residual unmapped terms. Full-dictionary back-translation confirmed semantic equivalence for 154 of 156 values (98.7%); the 2 exceptions were rare specimen-type variants that were refined. The verify_no_chinese.py script confirmed that zero CJK characters remained in the English-language deposit files. Across the deposited case-level file, these 156 dictionary entries account for the translation of 1,240 episode records spanning all 8 value domains.

### De-identification and *k*-anonymity

After de-identification, the *k*-anonymity scan on the year-month-coarsened public release found no quasi-identifier combination of (age band, sex, ward, admission month) with fewer than 5 episodes. No record suppression was required, because the minimum cell size was 7 (occurring in a single ward-month combination in the neonatology unit). All direct identifiers (names, MRNs, clinician names, and bed numbers, including temporary transfer assignments) were confirmed absent by an automated scan.

### FAIR assessment

Table 4 presents the FAIR Data Maturity Model assessment. Of the 15 evaluated indicators, 13 were scored as fully met and 2 as partially met [semantic interoperability with external ontologies (NHSN site cross-references provided but not full SNOMED-CT coding) and long-term preservation (Zenodo provides 10-year guaranteed retention but not indefinite archival)]. No indicator was scored as unmet.

### Machine-learning benchmark

**Fig. 4.**
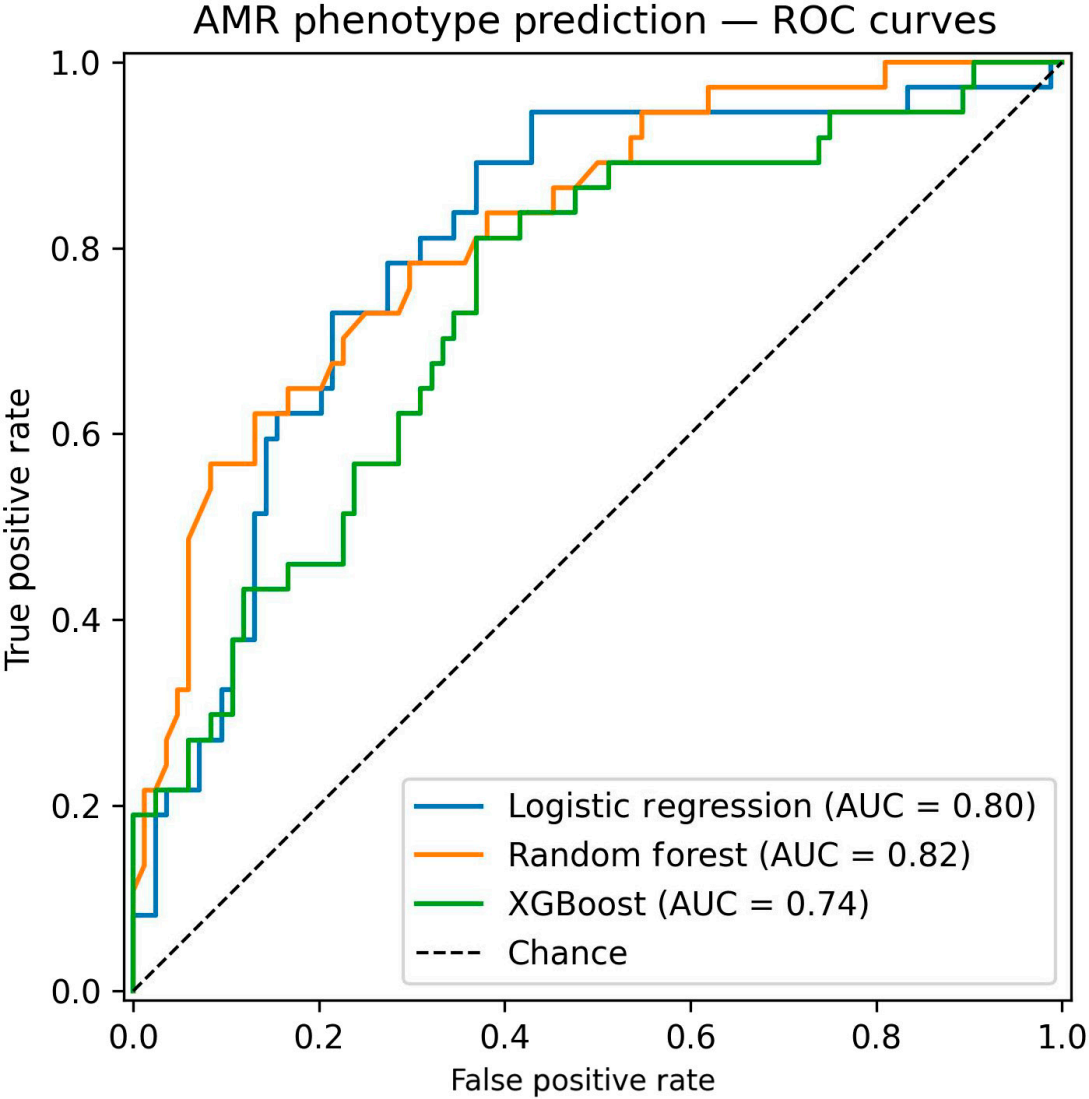
Receiver operating characteristic (ROC) curves for the 3 classifiers on the binary AMR phenotype-prediction task, evaluated on the held-out test set (*n* = 121; AMR prevalence 30.6%, 37/121) of the culture-positive subset. Areas under the curve were 0.82 for random forest, 0.80 for logistic regression, and 0.74 for XGBoost; the dashed diagonal denotes chance performance (AUROC = 0.50). Total length of stay (admission to discharge) was excluded from the feature set to prevent information leakage. AMR, antimicrobial resistance; AUROC, area under the receiver operating characteristic curve; ROC, receiver operating characteristic.

On the held-out test set (*n* = 121; AMR prevalence 30.6%, 37/121) of the culture-positive subset, the 3 classifiers achieved moderate discrimination for binary AMR prediction (Table 5 and Fig. 4). Random forest achieved the highest discrimination [AUROC 0.82, 95% CI 0.74 to 0.89; area under the precision–recall curve (AUPRC) 0.69, 95% CI 0.52 to 0.83] and, at the default 0.50 threshold, favored specificity (0.94) over sensitivity (0.49). Logistic regression (AUROC 0.80, 95% CI 0.71 to 0.88; AUPRC 0.62, 95% CI 0.45 to 0.78) was more sensitive (0.78) but less specific (0.70), while XGBoost was intermediate (AUROC 0.74, 95% CI 0.64 to 0.83; AUPRC 0.60). All 3 AUPRC values exceeded the no-skill baseline (prevalence 0.31), and F1-macro ranged from 0.66 to 0.73. These values indicate that the deposited dataset carries a learnable signal for a clinically relevant prediction task using standard methods, while reflecting the modest sample size and the deliberately conservative, unoptimized baseline; the contrast between the sensitivity-oriented logistic-regression operating point and the specificity-oriented tree ensembles also illustrates the threshold trade-offs available to downstream users.

### Reproducibility verification

Two independent executions of run_all.py on the deposited dataset produced byte-identical CSV outputs and numerically identical ML metrics (absolute differences < 1e−5 in all reported values). Total execution time was 2 min 47 s on a standard laptop (Intel i7, 16 GB RAM, Python 3.11). The pipeline requires only 6 Python packages (pandas, numpy, scipy, openpyxl, scikit-learn, and xgboost) with pinned versions specified in requirements.txt and a conda environment.yml. To further strengthen reproducibility, we provide a Dockerfile that pins Python 3.11 and all dependencies so that run_all.py executes in a byte-identical environment on any host, and the project is mirrored on a public GitHub repository (https://github.com/Griseldaoipl/hai-fair-pipeline) with a continuous-integration workflow that runs the unit tests and executes run_all.py on every commit. The pipeline follows semantic versioning; each release is archived as a Zenodo version with its own DOI, while the concept DOI resolves to the latest version, and backward-incompatible schema changes are reserved for major versions and documented in a CHANGELOG.

## Discussion

### Principal findings

This study presents what is, to the best of our knowledge, the first publicly available, FAIR-compliant, patient-level HAI surveillance dataset from a Chinese district-level hospital, together with a reproducible bilingual data pipeline and open-source code that enables replication at similar institutions. The primary contribution is the dataset itself: 1,240 HAI episodes with 57 variables, of which 604 culture-positive episodes carry AMR markers, with high completeness (>95% for 54 of 57 variables), validated bilingual translation (98.7% semantic equivalence across the full dictionary), and validated risk-factor extraction (sensitivity 0.92 to 1.00, specificity >0.97). The dataset fills a documented void in Chinese clinical-AI research infrastructure, where, despite rapid digitization of hospital information systems, machine-readable HAI surveillance data remain largely inaccessible to the research community. The proof-of-concept benchmark (AUROC up to 0.82 for AMR prediction using standard methods) demonstrates that the dataset contains sufficient signal for a clinically relevant prediction task and establishes a transparent baseline for future algorithm development.

### Comparison with existing HAI data resources

To the best of our knowledge, this is the first publicly available, FAIR-compliant HAI surveillance dataset from a Chinese district-level hospital. To support this claim, we searched Zenodo, figshare, Dryad, PhysioNet, and PubMed (April 2026) using combinations of the terms “healthcare-associated infection”, “nosocomial”, “surveillance”, “China”, and “open data”, and identified no patient-level, FAIR-compliant HAI dataset from a Chinese district-level hospital. Existing open HAI resources are predominantly from high-income settings: the MIMIC-IV database (Beth Israel Deaconess Medical Center, USA) includes infection episodes but is not structured as a dedicated HAI surveillance resource [17]; the OUTPAT-RESIST dataset provides community-acquired resistance patterns from European primary care [18]; and the CHINET annual reports publish aggregated resistance proportions without patient-level data [19]. Beyond geography, the present resource differs along several axes—care level (district rather than tertiary), granularity (patient-level episodes rather than aggregates), explicit linkage of risk factors, microbiology, and AMR markers, source language (bilingual Chinese-English with preserved source terms), and documented preprocessing provenance—which together constitute its methodological and infrastructural contribution rather than merely its existence as a new dataset.

### Research questions newly enabled by this dataset

Beyond filling a geographic and healthcare-system gap, public availability of this dataset enables several categories of research that were previously infeasible without direct hospital-data access. First, the bilingual structure (Chinese source terms mapped to English controlled vocabularies) supports cross-linguistic research on clinical natural-language processing, terminology harmonization, and translation-quality assessment for non-English EMR systems. Second, the 39-month longitudinal span with month-level resolution enables time-series analysis, seasonal-pattern detection, and temporal-validation strategies that are often absent from cross-sectional clinical datasets. Third, the combination of rich risk-factor annotations (19 validated binary flags) with AMR phenotype labels supports hypothesis generation on resistance determinants, risk-stratification model development, and feature-importance studies without access to restricted full-EMR data. Fourth, the documented pipeline and open code provide a reproducible template for other Chinese hospitals to convert their own surveillance archives into FAIR-compliant resources, potentially enabling multi-center federated learning or meta-analysis if multiple institutions adopt comparable schemas.

Importantly, the dataset’s modest size (*n* = 1,240) and single-institution origin constrain its use as a direct clinical-deployment resource: External validation on independent cohorts remains essential before any model trained on these data could be considered for real-world application. The dataset’s value lies not in immediate clinical deployment but in lowering barriers to algorithm prototyping, benchmark establishment, and methodological research for investigators without hospital partnerships or data-use agreements.

### Lessons for data-pipeline design in Chinese hospitals

Three design decisions were critical for pipeline robustness. First, the “fail-loud” principle—raising exceptions on unmapped headers or untranslated values rather than silently dropping them—ensured that format drift was detected at the point of introduction rather than propagating as silent data loss. Second, preserving the raw composite risk-factor string alongside the decomposed binary flags enabled retrospective auditing when new factor variants were discovered in subsequent months. Third, the cross-monthly reconciliation check (source row count = output row count for every month) provided a simple but powerful integrity guarantee that caught 2 early-stage bugs during development. We recommend these 3 principles—fail-loud mapping, raw-field preservation, and cross-source reconciliation—as minimum standards for any hospital data pipeline intended to produce research-grade output [7,20].

### Dataset utility and benchmark interpretation

The AMR-prediction AUROC of 0.74 to 0.82 indicates that the deposited dataset carries a learnable but moderate signal for this clinically relevant task. These values sit within the range reported for AMR-prediction models in the literature (AUROC 0.74 to 0.92 [4,5]), toward its lower-to-middle end, as expected for a single-center dataset of modest size analyzed with deliberately unoptimized, default-hyperparameter models and no feature engineering. We note a methodological point of broader relevance: An earlier version of this benchmark reported substantially higher AUROC, and on re-examination, we found that total length of stay—an admission-to-discharge variable not available at the prediction time point—had introduced post-outcome information (data leakage). After excluding this variable and restricting the feature set to information available at or before infection diagnosis, the corrected and fully reproducible results are those reported here. Researchers using the dataset should be able to exceed these baselines with hyperparameter tuning, feature engineering, and temporal-validation designs [8].

### Generalizability and pipeline portability

The single-institution origin of this dataset has important implications for generalizability. HAI epidemiology varies substantially across geographic regions, hospital tiers, patient populations, and antimicrobial-stewardship practices. The pathogen distribution observed in this district-level hospital in southwestern China (predominantly *Acinetobacter baumannii*, *Escherichia coli*, and *Klebsiella pneumoniae*) may differ from tertiary referral centers, specialized hospitals, or institutions in other provinces with different referral patterns, prescribing cultures, or resistance ecologies. Likewise, the AMR prevalence (30.6% among culture-positive episodes) reflects local antimicrobial selection pressure and infection-control practices during 2023–2026 and should not be extrapolated to other settings without validation. Prediction models trained on this dataset should undergo external validation before deployment, and performance degradation is expected when they are applied to hospitals with substantially different case mixes, surveillance protocols, or laboratory practices.

The pipeline methodology, however, is designed for portability. The 5 data-engineering challenges addressed—format drift, bilingual translation, composite-field decomposition, denominator reconciliation, and de-identification—are not unique to this institution but reflect systemic characteristics of Chinese hospital surveillance data: decentralized Excel-based reporting, mixed Chinese-English terminology, free-text risk-factor documentation, and fragmented information systems. Hospitals using different HIS vendors or templates will encounter different column names and value sets, but the architectural pattern (iterative schema detection, exception-driven mapping maintenance, deterministic transformation stages, and validation checkpoints) transfers directly, and the maintained JSON mapping files and translation dictionaries serve as adaptable starting templates rather than requiring development from scratch. For an institution with comparable Excel-based surveillance exports, we estimate approximately 20 to 40 person-hours of one-time set-up: schema inventory and header-mapping construction (~8 to 15 h, scaling with the number of template variants), extension of the translation dictionary to local terminology (~6 to 12 h), risk-factor glossary curation (~4 to 8 h), and validation on a stratified sample (~4 to 6 h); institutions using different HIS or LIS vendors, or with less standardized free-text documentation, will require additional effort, chiefly in the translation and risk-factor glossaries. Portability requirements include Python 3.11+ with standard scientific libraries, source data in Excel or CSV format with identifiable header rows, maintenance of institution-specific mapping files for local terminology, and de-identification aligned with local regulatory requirements.

### Semantic interoperability and future ontology mapping

The current dataset achieves partial semantic interoperability: All Chinese categorical values are translated to English controlled vocabularies, and infection-site categories are cross-referenced to NHSN standard definitions to enable comparison with U.S. HAI surveillance data. Full ontology mapping to SNOMED-CT, ICD-10-CM, LOINC, or other internationally recognized terminologies was not completed in this initial release, owing to resource constraints and the complexity of many-to-many mappings between Chinese clinical terms and international code systems. Future versions will prioritize 3 enhancements: mapping pathogen names to NCBI Taxonomy identifiers and SNOMED-CT organism codes, dual-coding infection sites using both NHSN definitions (current) and ICD-10-CM diagnosis codes (planned), and mapping risk-factor categories to SNOMED-CT clinical findings or procedures where direct equivalents exist. Interoperability with existing research-data ecosystems will be pursued along 2 further tracks: restructuring the patient-level table to the OMOP Common Data Model (mapping episodes, microbiology, and risk factors to the Condition, Measurement, Drug_Exposure, and Observation domains) to support Observational Health Data Sciences and Informatics (OHDSI)-style network studies; and exposing records as HL7 FHIR resources (Condition, Observation, and Specimen) for interoperability with FHIR-native clinical systems. We welcome collaboration with ontology experts and international HAI surveillance consortia, and the preserved bilingual structure facilitates such mapping. Enhanced interoperability will be released as versioned Zenodo updates with DOI versioning to preserve reproducibility of analyses based on earlier versions.

### Limitations

This study has several limitations. First, the pipeline was validated in a single hospital’s surveillance system; other Chinese hospitals may use different information-system vendors with different export formats, requiring adaptation of the header-mapping and translation dictionaries. Second, the dataset is single-center (*n* = 1,240 episodes), which limits statistical power for rare outcomes and prevents assessment of between-hospital heterogeneity. Third, comorbidity data were recorded as free-text Chinese rather than ICD-coded, so we could not derive validated comorbidity indices; the 19 binary risk-factor flags are a pragmatic substitute. Fourth, patient-day denominators were unavailable for 2023, restricting rate-based analyses to 28 months.

Fifth, the risk-factor decomposition, although validated against human expert annotations (sensitivity 0.92 to 1.00, specificity >0.97), depends on the completeness of the source Chinese free-text field: If infection-control staff did not document a risk factor that was clinically present, the derived flag reflects the documentation gap rather than true clinical status. The validation assessed decomposition accuracy (was the documented text correctly parsed?), not documentation completeness (were all clinically relevant factors documented?). We also note that the deterministic substring-matching approach, although well suited to the standardized, closed-vocabulary free-text of this surveillance system, may require augmentation for hospitals with less standardized or narrative documentation; future versions could incorporate clinical named-entity recognition or fine-tuned transformer-based language models [for example, Chinese clinical-domain bidirectional encoder representations from transformers (BERT) variants], with the present rule-based flags serving as a validated reference standard for training and evaluation. Sixth, the ML benchmark used a random rather than temporal train-test split, which may overestimate performance where temporal trends or changes in laboratory practice create distribution shift; the modest test set (*n* = 121) also yields wide CIs (AUROC interval widths of roughly 0.18 units), so the results should be read as a proof-of-concept that the dataset contains learnable signal rather than as validated model performance. Seventh, although de-identification followed HIPAA Safe Harbor precedent (*k* ≥ 5) and achieved *k* ≥ 7 for all quasi-identifier combinations, residual re-identification risk cannot be entirely eliminated for individuals with highly distinctive presentations in a small hospital during a known time window; this risk is inherent to small-scale clinical datasets and is balanced against the public-health value of data sharing. Finally, the benchmark uses internal validation only; external validation on independent datasets is required before any clinical-deployment consideration [8].

## Conclusion

We present a reproducible, open-source data pipeline that converts raw Chinese-language HAI surveillance records into a FAIR-compliant, AI-ready benchmark dataset. The pipeline, codebook, and deposited data (DOI: 10.5281/zenodo.20725167) are freely available for reuse. We demonstrate dataset utility through an AMR-prediction benchmark (AUROC up to 0.82, leakage-corrected). This approach is transferable to other Chinese district hospitals and provides a template for converting institutional surveillance archives into research-grade, machine-readable resources aligned with the FAIR principles and TRIPOD+AI reporting standards.

## Ethical Approval

This study was approved by the Ethics Committee of the People’s Hospital of Chongqing Hechuan (approval number: HX-2025-009). The committee waived individual informed consent because the study used de-identified surveillance data collected as part of routine hospital infection-control activities.

## Data Availability

The de-identified dataset (cases_long_en.csv, department_monthly_en.csv, department_period_en.csv), the bilingual codebook (codebook_en.csv), and the complete analysis pipeline are deposited in Zenodo under CC-BY 4.0 (data) and MIT (code) licenses. DOI: 10.5281/zenodo.20725167. The repository includes SHA-256 checksums, Git commit provenance, an environment specification (requirements.txt and environment.yml), the risk-factor decomposition scoring script together with the per-flag validation results (Cohen’s κ and per-flag sensitivity, specificity, and positive predictive value), and a single-command driver (run_all.py) that reproduces all results. The pipeline is additionally maintained on a public GitHub repository (https://github.com/Griseldaoipl/hai-fair-pipeline), with tagged releases archived to Zenodo. The raw per-episode expert annotations underlying the risk-factor validation are available from the corresponding author on reasonable request, subject to institutional data-sharing approval.
