# Supplementary material for: From Raw Hospital Records to an AI-Ready Surveillance Dataset: A FAIR-Compliant Data Pipeline for Healthcare-Associated Infection Research in a Chinese District Hospital
Source: Comput Struct Biotechnol J. 2026 Jul 27;35(2):0185. doi: 10.34133/csbj.0185 (PMC13402722; doi:10.34133/csbj.0185)
Supplement: Supplementary 1 — Fig. S1 Tables S1 and S2 [file csbj.0185.f1.zip › SupplementaryTable_S1.docx]

**Supplementary Table S1. Dataset Variable Summary by Domain (57 Variables)**

| **Domain** | **Count** | **Key Variables** | **Description** | **Completeness** | **AI Relevance** |
| --- | --- | --- | --- | --- | --- |
| Demographics | 4 | age_years, sex, admission_date, discharge_date | Patient age, sex, hospitalization dates | 100% | Risk stratification, demographic patterns |
| Infection Characteristics | 6 | infection_site, infection_date, days_to_infection, infection_type | HAI diagnosis, onset timing, site, type, outcome | 100% (outcome 97.3%) | Target variables, temporal features |
| Clinical Context | 6 | infection_dept, length_of_stay_days, icu_days, ventilator_days | Department, length of stay, ICU exposure | 88-100% | Severity proxies, healthcare utilization |
| Microbiology | 4 | micro_positive, specimen_type, pathogen, pathogen_count | Culture results, specimen source, organisms | 100% | AMR prediction, pathogen models |
| Risk Factors | 19 | 19 binary flags (rf_*): bedridden, catheters, immunosuppression, malignancy | Predisposing conditions from Chinese free-text | 100% | High-dimensional ML features |
| Surgical | 3 | incision_grade, surgery_start, surgery_end | Incision classification, operative timing | 32% (surgical only) | SSI prediction |
| Surveillance Metadata | 5 | year, month, report_month, surveillance_ward, report_status | Temporal identifiers, reporting status | 100% | Temporal validation, trend analysis |
| Aggregate Denominators | 10 | dept_patient_days, dept_infection_count, hospital_wide_rate | Monthly/quarterly department and hospital summaries | 100% | Incidence rates, burden assessment |
