# Supplementary material for: From Raw Hospital Records to an AI-Ready Surveillance Dataset: A FAIR-Compliant Data Pipeline for Healthcare-Associated Infection Research in a Chinese District Hospital
Source: Comput Struct Biotechnol J. 2026 Jul 27;35(2):0185. doi: 10.34133/csbj.0185 (PMC13402722; doi:10.34133/csbj.0185)
Supplement: Supplementary 1 — Fig. S1 Tables S1 and S2 [file csbj.0185.f1.zip › Supplementary_Table_S2_FAIR.docx]

**Supplementary Table S2.** FAIR Data Maturity Model assessment of the deposited dataset.

*Each of the 15 core indicators of the RDA FAIR Data Maturity Model (2020) applicable to a clinical dataset was independently scored by two authors (XP and DH) on a three-level rubric (fully met = 1.0, partially met = 0.5, not met = 0.0), with discrepancies reconciled by discussion. Partially met indicators are shown in red.*

| **Principle** | **ID** | **Indicator** | **Score** | **Status** | **Evidence** | **Deposit reference** |
| --- | --- | --- | --- | --- | --- | --- |
| Findable | **F1** | Data assigned a globally unique and persistent identifier | 1.0 | Fully met | Dataset has a Zenodo DOI (10.5281/zenodo.20725167), a globally unique persistent identifier. | DOI 10.5281/zenodo.20725167; README.md citation |
| Findable | **F2** | Data described with rich metadata | 1.0 | Fully met | Full variable-level metadata provided for every file and field. | data/codebook_en.csv; data/codebook_en.md |
| Findable | **F3** | Metadata clearly and explicitly include the identifier of the data | 1.0 | Fully met | Zenodo record metadata and README embed the DOI alongside the described files. | README.md; Zenodo record |
| Findable | **F4** | Data registered/indexed in a searchable resource | 1.0 | Fully met | Deposited in Zenodo and indexed by DataCite, making the record discoverable. | Zenodo / DataCite index |
| Accessible | **A1** | Data retrievable by identifier using a standardised protocol | 1.0 | Fully met | Record and files retrievable over HTTPS via the resolved DOI. | Zenodo HTTPS; DOI resolver |
| Accessible | **A1.1** | Protocol is open, free and universally implementable | 1.0 | Fully met | HTTPS is open, free and universally implementable. | Zenodo access protocol |
| Accessible | **A2** | Metadata accessible even when the data are no longer available | 1.0 | Fully met | Zenodo retains DOI-linked metadata (tombstone) independent of file availability. | Zenodo preservation policy |
| Interoperable | **I1** | Data use a formal, accessible, shared, broadly applicable knowledge representation (semantic interoperability) | **0.5** | Partially met | Infection sites and pathogens map to a controlled internal vocabulary with cross-references to NHSN standard terminology; full SNOMED-CT / ICD-10-CM coding not yet implemented (resource constraints). | code/translation_maps.py; data/codebook_en.csv |
| Interoperable | **I2** | Data use vocabularies that themselves follow FAIR principles | 1.0 | Fully met | Categorical values normalised to a documented, versioned English vocabulary released with the deposit. | code/translation_maps.py |
| Interoperable | **I3** | Data include qualified references to other (meta)data | 1.0 | Fully met | Codebook links each variable to its file and to the department/site/pathogen crosswalks. | data/codebook_en.csv; data/codebook_en.md |
| Reusable | **R1** | Data richly described with a plurality of accurate and relevant attributes | 1.0 | Fully met | 57 documented variables per episode including temporal, departmental, microbiological, device and outcome attributes. | data/cases_long_en.csv; data/codebook_en.csv |
| Reusable | **R1.1** | (Meta)data released with a clear and accessible data usage license | 1.0 | Fully met | Code released under MIT; data released under CC-BY 4.0, both stated explicitly. | LICENSE; README.md |
| Reusable | **R1.2** | (Meta)data associated with detailed provenance | 1.0 | Fully met | Provenance keys (YYYY-MM source_file) and a documented six-stage pipeline describe data origin and processing. | code/run_all.py; code/stage1_ingestion.py..stage6_deidentification_qa.py |
| Reusable | **R1.3** | (Meta)data meet domain-relevant community standards (long-term preservation) | **0.5** | Partially met | Zenodo guarantees 10-year retention with DOI persistence; indefinite archival (e.g., national archive) not yet established. | Zenodo retention policy |
| Reusable | **R1.4** | Data deposited in a trusted, certified repository | 1.0 | Fully met | Deposited in Zenodo, a CERN-backed general-purpose research repository. | Zenodo record |

**Summary.** 13 of 15 indicators were fully met and 2 were partially met (I1, semantic interoperability with external ontologies; and R1.3, indefinite long-term preservation), giving a mean maturity score of 0.93. No indicator was unmet.

**Independent automated cross-check.** The published record was additionally evaluated with the automated F-UJI tool (v3.5.1; FsF metric specification v0.8), which returned an overall FAIR score of 80% (“moderate”; 21/26), with Findable 6/7, Accessible 6/7, Interoperable 4/6, and Reusable 5/6. The one automated metric scored as not met (machine-actionable semantic vocabularies) is consistent with the I1 semantic-interoperability limitation recorded above. Full report: F-UJI JSON deposited with the record (DOI: 10.5281/zenodo.20725167).
