# Supplementary figures and images for: From Raw Hospital Records to an AI-Ready Surveillance Dataset: A FAIR-Compliant Data Pipeline for Healthcare-Associated Infection Research in a Chinese District Hospital
Source: Comput Struct Biotechnol J. 2026 Jul 27;35(2):0185. doi: 10.34133/csbj.0185 (PMC13402722; doi:10.34133/csbj.0185)

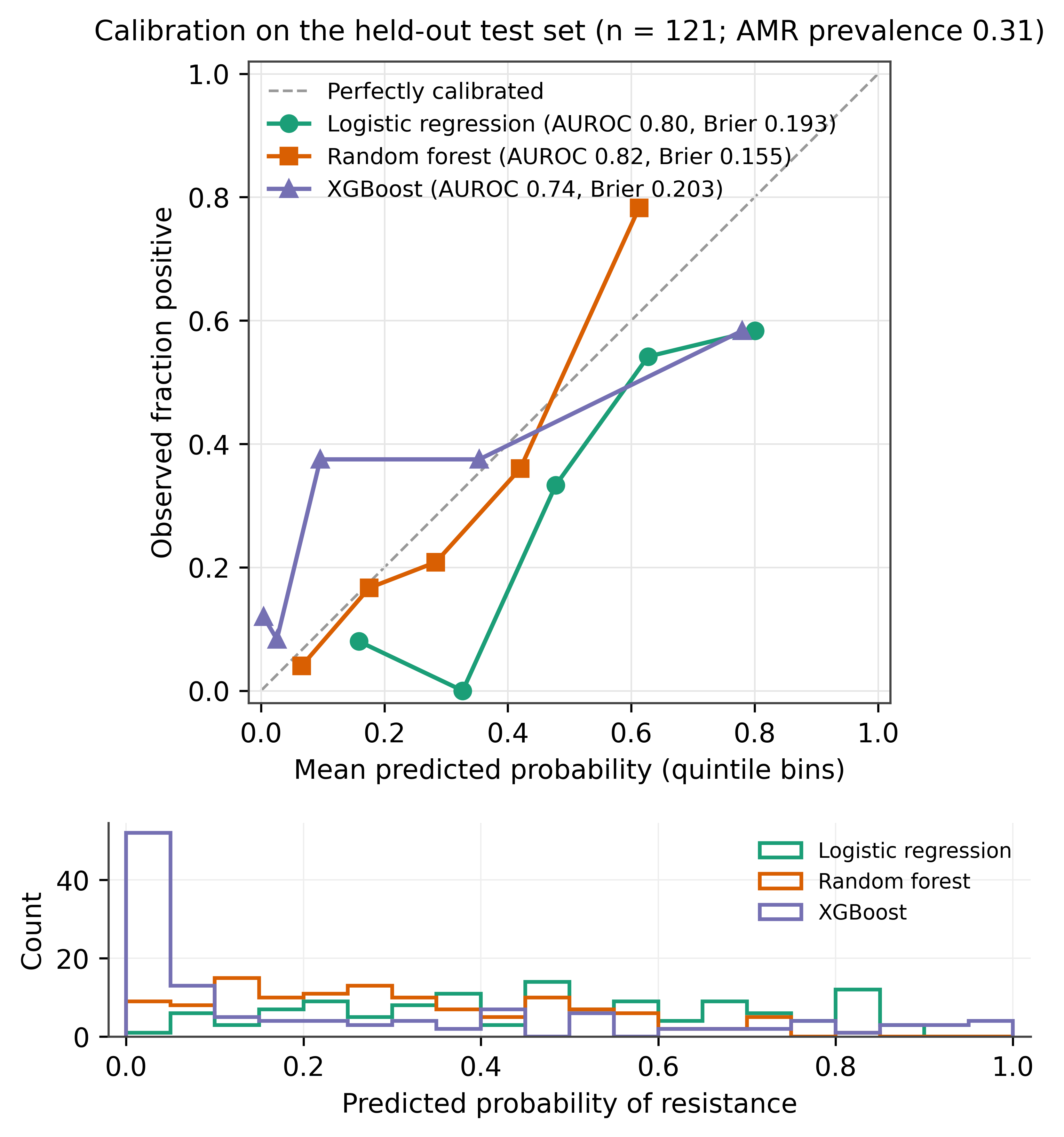

Supplement: Supplementary 1 — Fig. S1 Tables S1 and S2 [file csbj.0185.f1.zip › Supplementary_Figure_S1_calibration.png]
